# Reconstructive Surgery for Severe Penile Inadequacy: Phalloplasty with a Free Radial Forearm Flap or a Pedicled Anterolateral Thigh Flap

**DOI:** 10.1155/2008/704343

**Published:** 2008-11-04

**Authors:** N. Lumen, S. Monstrey, P. Ceulemans, E. van Laecke, P. Hoebeke

**Affiliations:** ^1^Department of Paediatric Urology and Urogenital Reconstruction, Ghent University Hospital, De Pintelaan 185, B-9000 Gent, Belgium; ^2^Department of Plastic, Reconstructive and Aesthetic Surgery, Ghent University Hospital, De Pintelaan 185, B-9000 Gent, Belgium

## Abstract

*Objectives*. Severe penile inadequacy in adolescents is rare. Phallic reconstruction to treat this devastating condition is a major challenge to the reconstructive surgeon. Phallic reconstruction using the free radial forearm flap (RFF) or the pedicled anterolateral thigh flap (ALTF) has been routinely used in female-to-male transsexuals. Recently we started to use these techniques in the treatment of severe penile inadequacy. *Methods*. Eleven males (age 15 to 42 years) were treated with a phallic reconstruction. The RFF is our method of choice; the ALTF is an alternative when a free flap is contraindicated or less desired by the patient. The RFF was used in 7 patients, the ALTF in 4 patients. Mean followup was 25 months (range: 4–49 months). Aesthetic and functional results were evaluated. *Results*. There were no complications related to the flap. Aesthetic results were judged as “good” in 9 patients and “moderate” in 2 patients. Sensitivity in the RFF was superior compared to the ALTF. Four patients developed urinary complications (stricture and/or fistula). Six patients underwent erectile implant surgery. In 2 patients the erectile implant had to be removed due to infection or erosion. *Conclusion*. In case of severe penile inadequacy due to whatever condition, a phalloplasty is the preferred treatment nowadays. The free radial forearm flap is still the method of choice. The anterolateral thigh flap can be a good alternative, especially when free flaps are contraindicated, but sensitivity is markedly inferior in these flaps.

## 1. INTRODUCTION

The biological male without penis or with severe penile inadequacy remains a major challenge to the reconstructive urological surgeon. A clear definition of severe penile inadequacy has not yet been established but we consider it as an insufficient penile length and function to obtain successful sexual intercourse. This implies that puberty must be finished and that the patient must be sexually active.

Next to congenital conditions, which resulted in inadequate penile development, traumatic events and medically indicated penile amputations, as well as failed reconstructions of congenital anomalies are also the main reasons for severe penile inadequacy.

An absent or inadequate penis is a devastating condition with significant psychological and physical impact. Although uncommon, it is a challenging condition to treat.

The different cosmetic and functional requirements for penile reconstruction are well known as follows. (i) The aesthetic appearance of the neophallus must be as normal as possible. (ii) The penile shaft must contain a urethra to allow voiding in a standing position and with a normal stream. (iii) The penile shaft must allow the implantation of a penile stiffener in order to allow intercourse. (iv) Morbidity of the donor area must be minimal with an easily concealed scar. Although phallic reconstruction is a complex surgical procedure, it is nowadays possible to fulfil most of the above-mentioned requirements using the new techniques developed in plastic and reconstructive surgery.

A large experience has been obtained at our centre with more than 350 female-to-male transsexuals who underwent a penile reconstruction. In this paper, our experience in phalloplasty using the free radial forearm free flap (RFF) or the pedicled anterolateral thigh flap (ALTF) is described for patients with severe penile inadequacy.

## 2. MATERIAL AND METHODS

A cohort of 11 patients (age: 15–42 years) who underwent phalloplasty at our institution was retrospectively analyzed. Median followup was 25 months (range: 4–49 months). All of them lost most of their functional penile tissues due to different conditions (Table 1). Emptying of the bladder was done through a urethral opening in 6 patients and by catheterisation through an appendico-vesicostomy in 5 patients. Two of these patients requested a urethral reconstruction for ejaculation, the other 3 preferred to keep their ejaculatory opening at the ventral aspect of the scrotum. All of the patients received psychological support and were given the opportunity to talk with previously operated transsexuals. Our method of choice for penile reconstruction is the RFF. One patient refused an RFF because he wanted to avoid a scar on the forearm and, therefore, chose in favour an ALTF. Three patients were not a good candidate for a free flap: two patients had a micropenis due to cloacal exstrophy in which previous corrective and reconstructive surgeries had altered the pelvic anatomy and vasculature. One patient underwent penile amputation because of penile necrosis after persistent priapism. This patient was treated first with an unsuccessful cavernosal-femoral shunt and later embolisation of the pudendal artery. In this case, vascular anatomy was uncertain as well and a free flap was contraindicated. Thus, seven patients were treated with the RFF and 4 patients with an ALTF.

### 2.1. Surgical technique

**Figure 1:**
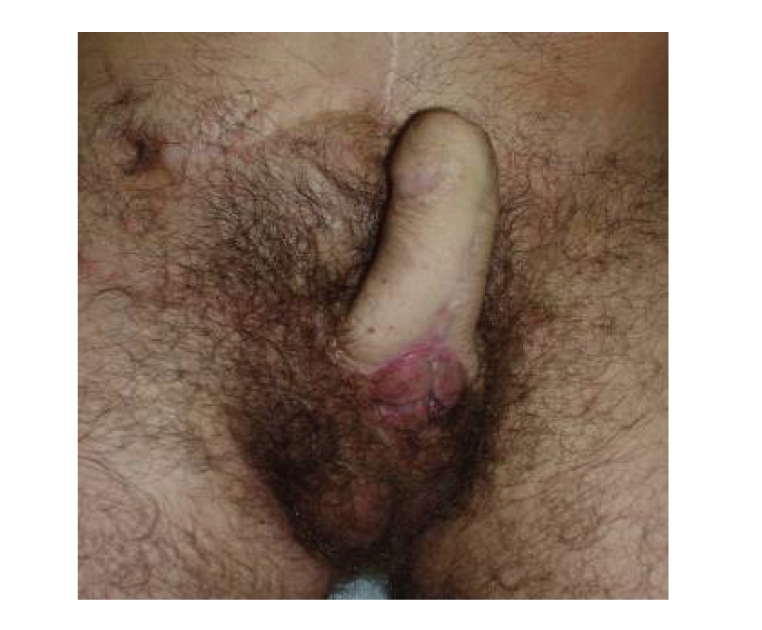
Incorporation of residual penile tissue at the base of the phallus.

Two operative teams are working simultaneously: the urological team (P. Hoebeke and N. Lumen) is preparing the acceptor area, while the flap is harvested by the plastic surgeons (S. Monstrey and P. Ceulemans). Depending on the underlying condition, any useful penile and cavernosal tissue is preserved in order to be incorporated at the basis of the phallus (Figure 1). The urethral stump, if available, is prepared for connection with the phallic urethra and, if available, a dorsal penile nerve is identified.

#### 2.1.1. Free radial forearm flap (Figure 2)

This flap is harvested from the forearm and shaped to a phallus using a tube-in-a-tube technique while being attached to the forearm by its vascular pedicle. A small skin flap and skin graft are used to create a corona and a sulcus to imitate a circumcised glans of the penis. The free flap is then transferred to the pubic area and after performing the urethral anastomosis, the radial artery is microsurgically connected end-to-side to the common femoral artery. The venous anastomosis is performed under microscopic magnification between the cephalic vein and the greater saphenous vein. One forearm nerve (N.cutaneus antebraci) is connected to the ilioinguinal nerve for protective sensation and the other nerve is anastomosed to the dorsal penile nerve for erogenous sensation.

#### 2.1.2. Anterolateral thigh flap (Figure 3)

This flap is a pedicled perforator flap supplied by the descending branch of lateral femoral circumflex artery. The perforator vessels are identified using Doppler-ultrasound just prior to incision. The lateral femoral cutaneous nerve is transsected after harvesting the flap. The flap is tunneled underneath the adductor muscles and then transferred to the pubic area. At this moment, the flap is shaped into a phallus using the tube-in-tube technique. Once at the pubic area, the urethral anastomosis is finished. Any tension on the pedicle must be avoided. The nerve is reattached to its stump using a subcutaneous tunnel above the adductor muscles.

The defect on the donor area is covered with split-thickness skin grafts harvested from the medial and anterior thigh. All patients receive a suprapubic urinary diversion postoperatively. The patients remain in bed during a one-week period after which the transurethral catheter is removed. One week later, the suprapubic catheter is clamped and voiding is started. It sometimes takes several days before good voiding is observed. The average admission period for the phalloplasty procedure is about 2,5 weeks. Tattooing of the glans can be performed after a 3 to 6 month period, before sensation is returned to the penis. For the implantation of a penile prosthesis, return of sensation to the top of the neophallus is required. This usually takes about one year. An AMS Ambico prosthesis using one cylinder is implanted. A median scrotal incision is used and the tract through the phallus is bluntly dilated. If possible, the base of the cylinder is fixed in the remnants of the corpora cavernosa. If not, the cylinder is attached to the ramus inferior ossis pubis with a nonresorbable suture. Evaluation of sensation and aesthetic appearance was done by questionnaire. Voiding was evaluated by uroflowmetrie and measurement of residual urine by echography. In case a stricture was suspected, urethrography was performed.

## 3. RESULTS

Mean dimensions of the flap (including urethra) were 15 by 14 cm.

A total flap survival was noticed in all patients, and there were no complications concerning the donor area. Eight of the eleven patients underwent urethral reconstruction. In 2 patients, this was only done for ejaculation through the phallus. In 8 patients, there was sufficient penile and/or cavernosal tissue to be incorporated at the base of the newly reconstructed phallus.

Of 8 patients in which a urethra was reconstructed, 4 patients developed urethral complications. A persistent fistula at the anastomosis of the neourethra to the native urethra developed in 3 patients (all treated by RFF), of which a concomitant urethral stricture was present in 2 patients. One ALTF-patient developed an isolated urethral stricture, also at the anastomosis site. In case of a fistula, surgical closure was needed and successful in all 3 patients. The urethral strictures were managed by end-to-end urethroplasty in one patient and by a two-stage urethroplasty in the other patient. After these secondary procedures, all patients (without appendico-vesicostomy) could void in a standing position.

**Figure 4:**
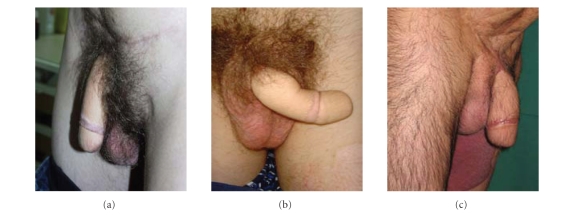
Excellent aesthetic appearence after RFF (a)-(b) and ALTF (c) phalloplasty.

Aesthetic appearance was excellent in 9 patients (Figure 4), which all expressed their extreme happiness with the result. In 2 patients, the aesthetic results were moderate. One patient was treated by an RFF and developed a hypertrofic scar causing a clear deformity of the phallus. A Z-plasty was used to solve this problem. The other patient was treated by an ALTF and suffered from a skin rash of unknown origin at the phallus but also at other places of his body.

All of the patients treated by RFFF report protective and erotic sensitivity in their phallus. The patients treated by ALTF have some sensation in the phallus supplied by the lateral femoral cutaneous nerve but markedly inferior compared to the RFF due to the fact that only protective sensation has been provided.

A penile prosthesis was implanted in 6 patients. Unfortunately, in 2 patients the erectile implant had to be removed because of infection. The other 4 patients report satisfactory sexual intercourse.

## 4. DISCUSSION

Reconstructive surgery for severe penile insufficiency is necessary because of the devastating effect on psychological and sexual function. Gender reassignment, as used in the past, has controversial results and is nowadays abandoned [1]. Reconstructive surgery with phalloplasty is available and generally used in female-to-male transsexuals. Phalloplasty procedures have followed advances made in plastic surgery. The development of microsurgical free-flap techniques made the first microsurgical phalloplasty possible using a free radial forearm flap [2]. The radial forearm flap has been widely accepted as the best donor site for penile reconstruction and is nowadays the golden standard in phalloplasty for female-to-male transsexuals [3–5]. This same technique can also be applied for severe penile insufficiency.

In this series, the RFF was the method of choice. No complications concerning flap survival or at the donor site were reported. Of the seven patients treated with RFF, the aesthetic appearance was good in 6 patients and moderate in 1 patient. Other series using the RFF in penile insufficiency also report encouraging results with a good aesthetic appearance and low donor site morbidity. The results of phalloplasty using radial forearm free flap in penile insufficiency are encouraging, the aesthetic appearance is good and donor morbidity is low [6, 7]. Erotic sensation was reported by all patients treated with RFF. Althougd subjective (a questionnaire was used), this finding is consistent with the work of Selvaggi et al. [8]. Coaptation of one of the cutaneous nerves of the flap with a remnant of the dorsal penile nerve seems to be essential in obtaining this result.

Nevertheless, other types of free flaps have been described: Djordjevic et al. [9] reported the musculocutaneous latissimus dorsi free flap, Sengezer et al. [10] suggested the osteocutaneous free-fibula flap, and N. Felici and A. Felici [11] described the free anterolateral thigh flap. They all report satisfactory results. The type of free flap that is used mostly depends on the personal preference and the experience of the plastic surgeon that is involved in phalloplasty.

In case of uncertain pelvic vasculature and anatomy, the use of a pedicled flap is preferred because it brings its own blood supply to the phallus. In our series, 2 patients had uncertain pelvic anatomy because of several previous reconstructive pelvic surgeries for cloacal exstrophy and one patient had uncertain pelvic vasculature because of previous shunting procedures and embolisation for persistent priapism. Possible flaps in these situations are the pedicled island groin flap (insensate) [12] or the pedicled anterolateral thigh flap [13]. In this series, one patient was a good candidate for RFF but he refused this technique because of the extensive scar at the forearm. This scar can be considered as a tell-tale sign of transsexualism. For this reason he preferred an ALTF. Although the aesthetic appearance was good in 3 patients and moderate in 1 patient, sensitivity remains a concern in these pediculated flaps. Although subjective (a questionnaire was used), they all reported less sensitivity compared to the patients treated with RFF. For this reason, an additional connection to the clitoris nerve should be considered.

The major drawbacks of phalloplasty are the urethral complications and the problems with the penile stiffeners. Urethral complication rate (stricture and/or fistula) was high but this is comparable to the large experience with transsexual phalloplasty [3]. Secondary procedures are needed to treat these complications in which the treatment of urethral strictures, especially, is challenging and difficult. Late occurrences of urethral stenosis are always possible because the skin urethra is prone to retract in the long term. If urethral reconstruction is not necessary, for example, in case of continent urinary diversion, it should not be performed unless the patient specifically asks for it.

Obtaining sufficient rigidity to allow penetration is extremely difficult because there is no good substitute for the unique erectile tissue of the penis. The RFFF and ALTF are too soft and, thus, implantation of a penile stiffener is needed for sexual intercourse. The implantation must be withheld until the urethra is free of strictures or fistulas and until the phallus is endowed with sufficient protective sensation. This usually takes 12 months. Sufficient protective sensation is needed in preventing breakdown and erosion of the stiffener. Despite all, explantations rates are high (20–50%) [3, 14, 15] and comparable to the 33,3% explantation rate in this series. One of the possible explanations for this is the less vascularised skin and subcutaneous tissue in the neophallus which can lead to diminished resistance against infection and perforation. Another reason can be the much more intensive use of the penile stiffener in comparison with mostly older and less active impotent men, with a higher chance of malfunction on the long term.

## 5. CONCLUSIONS

Due to the devastating impact on the psychological and sexual function, penile reconstruction of severe penile inadequacy is needed. Today, penile reconstruction using phalloplasty is available. A free flap, such as the radial forearm free flap, is the method of choice because of good aesthetic results, low donor site morbidity, and excellent erogenous sensitivity. In case a free flap is contraindicated, a pedicled flap, such as the anterolateral thigh flap, should be used. This flap has comparable aesthetic results, but sensitivity is a major concern in this flap.

Urinary complications and problems with penile stiffeners are frequent and patients must be informed about these possible complications. Despite this, phalloplasty is a valuable treatment option for severe penile insufficiency.

## Figures and Tables

**Figure 2:**
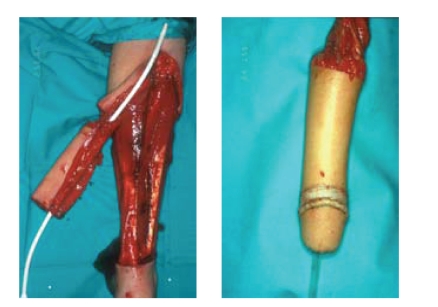
The radial forearm free flap using the tube-in-a-tube principle for creation of the neourethra.

**Figure 3:**
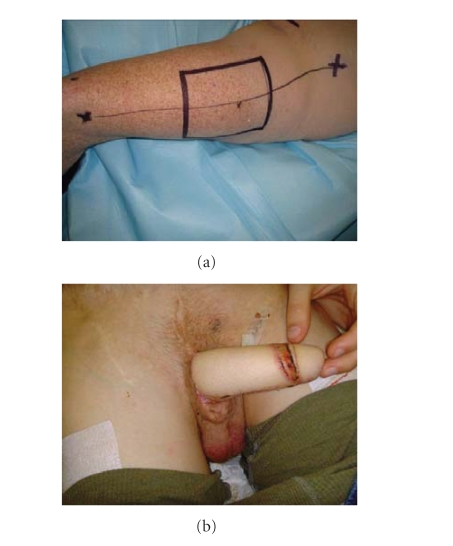
The anterolateral thigh flap. (a) Preoperative: the vascular pedicle is marked at the middle using Doppler-ultrasound. (b) Postoperative.

**Table 1:** Patients' characteristics.

Patient	indications	type of phalloplasty	age (years)
1	shrivelled penis—infected penile stiffener	anterlateral thigh flap	42
2	shrivelled penis—bladder exstrophy	radial forearm free flap	23
3	shrivelled penis—bladder exstrophy	radial forearm free flap	16
4	penile amputation—epitheloid sarcoma	radial forearm free flap	15
5	crippled penis—hypospadias	radial forearm free flap	20
6	shrivelled penis—bladder exstrophy	radial forearm free flap	15
7	penile necrosis—traffic accident	radial forearm free flap	32
8	shrivelled penis—cloacal exstrophy	anterlateral thigh flap	16
9	shrivelled penis—cloacal exstrophy	anterlateral thigh flap	16
10	micropenis—partial androgen insensitivity syndrome	radial forearm free flap	30
11	penile necrosis—embolisation for priapism	anterlateral thigh flap	38
